# Obstructive Sleep Apnea in Patients With and Without Diabetic Retinopathy: An Observational Study and Comparative Analysis

**DOI:** 10.7759/cureus.103775

**Published:** 2026-02-17

**Authors:** Kavita R Bhatnagar, Kiran Yadav, Kirti Jaisingh, Seema Meena, Naveen Dutt

**Affiliations:** 1 Ophthalmology, All India Institute of Medical Sciences, Jodhpur, IND; 2 Pulmonary and Critical Care Medicine, All India Institute of Medical Sciences, Jodhpur, IND

**Keywords:** apnea-hypopnea index, diabetic retinopathy, epworth sleepiness scale, obstructive sleep apnea, polysomnography

## Abstract

Introduction

Obstructive sleep apnea (OSA) causes intermittent hypoxia that may amplify retinal ischemia, accelerating the onset and progression of diabetic retinopathy (DR), specifically proliferative diabetic retinopathy (PDR). This study investigates the association between OSA and DR, including PDR, in an Indian cohort of type 2 diabetes mellitus (T2DM) patients.

Methods

In a prospective observational study conducted from October 2022 to December 2023 at a tertiary care centre, 85 T2DM patients (45 with DR, 40 without DR) underwent OSA screening using the Epworth Sleepiness Scale (ESS). High-risk patients (ESS ≥10) underwent polysomnography (PSG), with OSA severity graded by Apnea-Hypopnea Index (AHI).

Results

High OSA risk significantly correlated with longer diabetes duration (p=0.033), elevated HbA1c (p=0.030), dyslipidemia (p=0.003), hypertension (p=0.005), neck circumference (p=0.02), and BMI (p<0.001). DR prevalence was higher in high-risk OSA patients (58.33% vs. 52.05%, p=0.686), with a trend toward PDR (57.14% vs. 42%, p=0.943). PSG in nine patients revealed a trend showing greater oxygen desaturation index (ODI) and AHI in the DR group, albeit without statistical significance (p>0.05).

Conclusion

Though OSA association with DR and PDR remains statistically inconclusive, the results are exploratory and hypothesis-generating. Screening for OSA in resource-limited, busy DR clinics can detect additional risk factors. The novel AHI-sleep parameter trends advocate for further studies to validate OSA as a modifiable risk factor.

## Introduction

Obstructive sleep apnea (OSA) is characterized by repetitive episodes of complete or partial upper airway collapse during sleep, leading to airflow cessation (apnea) or reduction (hypopnea), resulting in arousal and hypoxia [1,2]. This chronic intermittent hypoxia may disrupt glycemic control by activating oxidative stress pathways and promoting the formation of advanced glycation end products (AGEs) [3]. These AGEs are implicated in various macrovascular and microvascular complications, including diabetic retinopathy (DR), a leading cause of vision loss in type 2 diabetes mellitus (DM) patients [4]. Studies suggest that OSA may be associated with increased microvascular damage, potentially increasing the risk of DR and its severe form, proliferative diabetic retinopathy (PDR) [5-7]. The hypoxia-driven mechanisms in OSA have been proposed to contribute to retinal ischemia and may influence the onset and progression of DR, including PDR, which is marked by neovascularization and severe visual impairment.

The severity of OSA is classified using the Apnea-Hypopnea Index (AHI) from polysomnography: mild (AHI 5-15 events/hour), moderate (AHI 15-30 events/hour), and severe (AHI >30 events/hour). Evidence indicates that high-risk OSA patients - those with elevated AHI-face a greater likelihood of developing DR and advancing to PDR due to intensified hypoxic stress [8,9]. Altaf et al. demonstrated that treating OSA with continuous positive airway pressure (CPAP) can mitigate PDR severity, suggesting a potential modifiable link between OSA and advanced DR [10]. Rahimy et al. showed that OSA not only increases the risk of progression of NPDR to PDR, but it also increases the risk of developing severe systemic complications like stroke and death in NPDR patients. On the other hand, there are studies that have found inconclusive evidence linking OSA and DR [11-14]. In developing countries like India, where DR prevalence is alarmingly high, identifying OSA as a contributing risk factor may have important implications for screening and management strategies [15].

Given OSA's potential to increase DR and PDR risk through hypoxia-mediated pathways, our study aimed to investigate this association in type 2 DM patients. We hypothesized that high-risk OSA patients exhibit a greater prevalence of DR, especially PDR, compared to those without OSA risk. By screening for OSA in DR patients, we seek to uncover an early, actionable comorbidity that could enhance treatment outcomes and reduce vision loss. This study underscores the critical need to further explore OSA as a potentially modifiable risk factor in the context of India's growing diabetic population.

## Materials and methods

This prospective observational study was conducted at a tertiary care centre in Western India from October 2022 to July 2024. It enrolled all type 2 diabetes mellitus patients visiting the retina clinic of the ophthalmology department during the study period. Approval was obtained from the Institutional Ethics Committee (AIIMS/IEC/2022/5232) dated 23/09/2022, and written informed consent was obtained from all study participants. The study adhered to the principles outlined in the Declaration of Helsinki.

With reference to the study conducted by Rakesh Kaswan et al. [16], and using the following formula, the sample size was calculated as follows: n=((Z(1-α/2) + Z(1-β))^2^ × 2Sp^2^) / μd^2^, where Sp^2^=(σ1^2^ + σ2^2^)/2; μd = μ1-μ2; Z(1-α/2) = 1.96 at 5% level of significance; Z(1-β) = 0.842 at β = 20%; σ1 = 1.2; σ2 = 1.9; μ1 = 7.5; and μ2 = 8.5. Considering this, we estimate a sample size of 40 in each group, i.e., 80 patients at 95% confidence interval and 90% power.

Of 310 screened patients, 237 met the eligibility criteria. However, 102 patients agreed to participate, and 85 completed the study (17 were lost to follow-up). Inclusion required a type 2 DM diagnosis and age >18, while exclusion criteria ruled out respiratory conditions including asthma, chronic obstructive pulmonary disease, interstitial lung disease, already diagnosed with OSA, and obvious upper airway anatomical abnormalities (e.g., severe deviated septum, large tonsils, or history of airway surgery). Apart from these, severe or decompensated cardiac conditions (congestive heart failure, recent acute coronary syndrome or myocardial infarction, Uncontrolled arrhythmias), other major comorbidities that could independently affect retinopathy or confound associations like end-stage renal disease or severe chronic kidney disease (e.g., eGFR <30 mL/min/1.73 m², advanced liver disease or cirrhosis, active malignancy or cancer treatment, pregnancy or planning pregnancy (due to physiological changes and safety concerns), neurological or neuromuscular disorders affecting respiration (e.g., stroke sequelae, myasthenia gravis), conditions impairing reliable retinal assessment (media opacities preventing adequate fundus visualization like dense cataract, vitreous hemorrhage), blindness or severe visual impairment unrelated to DR, substance abuse (e.g., alcohol or drug dependence) that could affect sleep or compliance, hypothyroidism (untreated or severe, as it can mimic/contribute to OSA symptoms), inability to comply with study procedures (e.g., cognitive impairment, refusal of polysomnography), were also excluded from the study. 

Demographic details of all patients were recorded, and a thorough medical history was obtained, including information on symptom onset, duration, and progression, medical illnesses, systemic risk factors, and family history of DM. Characteristics related to OSA, such as age, gender, body mass index (BMI), neck circumference, smoking and alcohol status, hypertension, and coronary heart disease, were also noted.

A comprehensive ophthalmic examination was carried out for all patients, including visual acuity (unaided and best corrected) using Snellen's chart, IOP with Goldmann applanation tonometer, central corneal thickness with the help of Sonomed A scan, detailed slit-lamp examination, and dilated fundus examination with both slit lamp biomicroscopy and indirect ophthalmoscopy. Central macular thickness was measured in all eyes with optical coherence tomography (OCT) (Topcon 3D OCT Maestro machine; Topcon, Livermore, California). The thickness of the peripapillary retinal nerve fibre layer was also measured in all eyes using the same OCT. All patients underwent wide-field colour fundus photography and Fundus fluorescein angiography on the wide-field Optos (Optos, Dunfermline, Scotland) machine.

Based on all the investigations, patients were grouped as DR (with diabetic retinopathy) or no DR (without diabetic retinopathy), according to the standard Early Treatment of Diabetic Retinopathy (ETDRS) classification. The dilated fundus examination and all retinal investigations were carried out by a single observer.

The risk of OSA was then assessed in all participants using a standard questionnaire, the Epworth Sleepiness Scale (ESS) questionnaire [17]. It consists of a total of eight questions, and a Likert scale is used to grade the responses from zero to three for all questions. A score of more than 10 is considered high risk for OSA.

Patients at high risk for OSA were referred to the Department of Pulmonary Medicine, specifically the sleep clinic, for polysomnography (PSG). The PSG setup involves the placement of several electrodes, viz. bilateral frontal, central, and occipital electrodes (EEG), surface chin and leg electromyogram (EMG), left and right eye electrooculogram (EOG), electrocardiogram lead II, audio and video recording for monitoring snoring, body position, and other abnormalities, nasal pressure transducer, oronasal thermal flow sensor, thoracic and abdominal respiratory effort, typically monitored with respiratory inductive plethysmography (RIP) belts, and pulse oximeter. Following the placement of electrodes, the patient is instructed to await calibration. Once calibration is complete, the patient is placed in a calm, dark, and quiet room to promote sleep. However, vigilance is maintained while monitoring the patient's PSG recordings and any physiological abnormalities, along with the video feed. The procedure concludes in the morning. A valid study mandates that the patient sleeps for at least two hours during the PSG. The severity of OSA is then categorized based on the AHI.

Several sleep parameters were recorded during PSG, such as AHI, lowest oxygen saturation, Oxygen Desaturation Index (ODI), sleep latency, sleep efficiency, and total sleep time.

Data was entered and analysed using the SPSS version 25 (IBM Inc., Armonk, New York) and Jamovi software. All nominal variables were described using counts and percentages and analysed using the Chi-squared or Fisher's exact test. All continuous variables were described using mean and standard deviation (SD) and analysed using the independent samples t-test. A p-value of <0.05 was considered statistically significant.

## Results

This study divided 85 type 2 DM patients into two main groups: 45 DM patients with DR were selected as cases, and 40 DM patients with no DR were taken as controls. There was a male predominance, with 55 male patients (64.7%) and 30 female patients (35.29%).

Amongst the DR patients, 25 patients had non-proliferative DR (NPDR), and 20 had proliferative DR (PDR). Amongst the NPDR patients, three had mild NPDR, 16 had moderate NPDR, three had severe NPDR, and three had very severe NPDR. Thirteen patients had diabetic macular edema.

ESS classified 14.1% patients (n=12) at severe risk. High OSA risk correlated significantly with diabetes duration (13.7±11.3 vs. 8.45±7.01 years, p=0.033), HbA1c (10.2±1.9% vs. 8.9±2.0%, p=0.030), total cholesterol (212±41.4 mg/dl vs. 186±43.4 mg/dl, p=0.053), low-density lipoprotein (LDL; 145±34.5 mg/dl vs. 115±36.7 mg/dl, p=0.009), LDL/high-density lipoprotein (HDL) ratio (2.25±0.93 vs. 3.09±0.60, p=0.003), hypertension (83.33% vs. 39.72%, p=0.005), neck circumference (15.8±0.97 vs. 14.8±0.97 cm, p=0.002) and BMI (29.5±6.73 kg/m2 vs. 24.1±4.36 kg/m2, p<0.001) (Table 1).

**Table 1:** Demographics, clinical and biochemical profile of OSA patients with the Epworth Sleepiness Scale OSA - obstructive sleep apnea; ESS - Epworth Sleepiness Scale Score; HbA1c - glycated hemoglobin; LDL - low-density lipoprotein; HDL - high density lipoprotein; BMI - body mass index; OHA - oral hypoglycemic agents

Risk factors	ESS > 10 (n=12)	ESS<10 (n=73)	p value
Age (yrs)	55.2±7.72	52.9±8.69	0.395
Gender (M/F)	8/4	47/26	0.878
Duration of diabetes (yrs)	13.7 ±11.3	8.45±7.01	0.033
HbA1c (%) (mmol/mol)	10.2± 1.9 (67-109)	8.9±2.0 (52-96)	0.030
Total cholesterol	212±41.4	186±43.4	0.053
LDL(mg/dl)	145±34.5	115±36.7	0.009
HDL(mg/dl)	47.3±7.92	53±10.6	0.078
Triglycerides(mg/dl)	170± 0.69	150± 0.98	0.170
LDL/HDL ratio	2.25± 0.93	3.09±0.60	0.003
24-hr protein (mg/day) (median)	186	108	0.387
Neck circumference (cm)	15.8± 0.97	14.8± 0.97	0.002
BMI (kg/m2)	29.5±6.73	24.1±4.36	<0.001
Heart diseases	1 (8.33%)	2 (2.74%)	0.396
Hypertension	10 (83.33%)	29 (39.72%)	0.005
Smoking	2 (16.66%)	14 (19.17%)	0.837
Alcohol	2 (16.66%)	14 (19.17%)	1.000
OHA	7 (58.33%)	59 (80%)	0.061
Insulin	5 (41.66%)	13 (17.8%)	

Heart diseases, 24-hour proteinuria, and insulin use were high in the high-risk OSA group but statistically insignificant. Smoking and alcoholism were not significantly associated (Table 1).

Though DR prevalence was higher in both high-risk and low-risk OSA patients, the difference between patients having DR and no DR was higher in high-risk patients, approximately 16% difference vs 4% difference in low-risk patients (OR=0.78, p=0.686). (Table 2)

**Table 2:** OSA as a risk factor for developing diabetic retinopathy DR - diabetic retinopathy; OSA - obstructive sleep apnea

Questionnaire	Score	DR group (n)	No DR group (n)	p-value
Epworth Sleepiness Scale score	>10(n=12)	7 (58.33%)	5 (41.66%)	0.686, odds ratio -0.776 (0.225-2.67)
	<10(n=73)	38 (52.05%)	35 (47.94%)	

There was a notable trend toward PDR (57.14% vs 42.85%, OR=1.04, p=0.943), though not statistically significant (Table 3).

**Table 3:** Risk of developing proliferative diabetic retinopathy and non-proliferative diabetic retinopathy in the presence of high- and low- risk of OSA PDR - proliferative diabetic retinopathy; NPDR - non-proliferative diabetic retinopathy; OSA - obstructive sleep apnea

Questionnaire	Score	PDR (n)	NPDR (n)	p-value
Epworth Sleepiness Scale score	>10(n=7)	4 (57.14%)	3 (42.85%)	0.943, odd ratio- 1.044
	<10(n=38)	16 (42.00%)	22 (57.89%)	

Of nine patients undergoing PSG, five had severe, two moderate, and two mild OSA. The DR group exhibited a higher median AHI (70.9 vs. 26.9, p=0.072), ODI (61.1 vs. 37.6, p=0.328), and lower oxygen saturation (75% vs. 82%, p=0.275), although the differences were not statistically significant (Table 4).

**Table 4:** Polysomnographic characteristics of patients according to the presence or absence of diabetic retinopathy PSG - polysomnography; DR - diabetic retinopathy; ODI - Oxygen Desaturation Index; AHI - Apnea-Hypoapnea Index; O2 - oxygen

PSG parameters	DR group (n=3)	No DR group (n=6)	p-value
ODI	61.1	37.6	0.328
AHI	70.9	26.9	0.072
Lowest O2 saturation (%)	75	82	0.275

AHI showed moderate negative correlations with total sleep time (rho=-0.1000, p=0.333) and a positive correlation with BMI (rho=1.000, p=0.333), though not significant. Sleep efficiency showed positive correlation with total sleep time (rho=0.500, p=1.000) and negative correlation with sleep latency (rho=-0.500, p=1.000), but the results were not statistically significant (Table 5).

**Table 5:** Correlation of polysomnography parameters with the Apnoea-Hypopnea Index (AHI) and sleep efficiency in type 2 diabetes mellitus patients PSG - polysomnography; AHI - Apnea-hypoapnea index; BMI - body mass index

PSG variables	Diabetic retinopathy (n=3)		No diabetic retinopathy (n=6)	
	Spearman’s rho	p-value	Spearman’s rho	p-value
Apnoea-Hypopnea Index (AHI)				
Total sleep time	-0.1	0.333	-0.371	0.497
BMI	1	0.333	0.6	0.242
Sleep efficiency				
Total sleep time	0.5	1	0.143	0.803
Sleep latency	-0.5	1	-0.429	0.419

## Discussion

This study investigated the prevalence and severity of obstructive sleep apnea (OSA) risk among type 2 diabetes mellitus (T2DM) patients, focusing particularly on its association with diabetic retinopathy (DR) and proliferative diabetic retinopathy (PDR) in an Indian tertiary care setting. We observed a higher proportion of diabetic retinopathy (58.33%) and proliferative diabetic retinopathy (57.14%) among participants classified as high risk for obstructive sleep apnea compared with those at low risk (52.05% DR and 42.0% PDR). However, these differences were not statistically significant (p=0.686 for DR and p=0.943 for PDR). While the findings demonstrate only a modest, non-significant trend, they are consistent with the proposed role of intermittent hypoxia and metabolic stress in microvascular dysfunction. These observations should be interpreted with caution and warrant further investigation in larger studies to clarify the potential relationship between obstructive sleep apnea risk and diabetic retinopathy, particularly in the Indian population, where the burden of diabetic eye disease is substantial.

Our results reinforce and extend prior research highlighting OSA as a potential risk factor for DR and PDR. Rahimy et al. showed a significantly elevated risk of PDR and diabetic macular edema in patients with NPDR and OSA, attributing it to hypoxia's role in retinal neovascularization [6]. Similarly, Altaf et al. identified a stronger OSA-PDR link in White Europeans (OR=2.3, p<0.01), though less pronounced in South Asians, possibly due to differing metabolic profiles [10]. Chang et al. reported a significant correlation between the severity of OSA and DR and diabetic macular oedema [11]. Our Indian cohort's findings-high-risk OSA linked to 57.14% of PDR cases-suggest a comparable hypoxia-driven risk, with 14.1% of patients classified as high-risk via ESS screening. Unlike Kaswan et al.'s 2021 Indian study (n=150), which found no significant OSA-DR correlation (p=0.62) using subjective tools alone, our inclusion of polysomnography (PSG) in a subset (n=9) revealed elevated oxygen desaturation index (ODI) and AHI in the DR group, supporting a heightened risk profile in high-risk OSA patients, particularly for PDR [17].

The small PSG sample (n=9) limited our ability to achieve statistical significance (e.g., AHI: 70.9 vs. 26.9, p=0.072), a constraint reflecting resource challenges in busy DR clinics. However, this does not diminish the clinical implication. Type 2 diabetic patients demonstrated a 14.1% high risk OSA prevalence, with severe cases (AHI >30) more common in the DR group. This aligns with Leong et al.'s 2016 meta-analysis, which noted hypoxia's plausible role in DR progression, especially PDR, despite variable statistical outcomes [2]. Our novel contribution lies in correlating AHI with sleep parameters (e.g., negative trend with sleep efficiency, rho=-0.314), suggesting that OSA-related sleep disruption may amplify DR and PDR risk, a finding underexplored in prior Indian studies. This hypoxia-sleep interplay could explain the elevated PDR prevalence in our high-risk OSA subgroup.

High-risk OSA's association with metabolic and anthropometric factors, longer diabetes duration (p=0.033), elevated HbA1c (p=0.030), dyslipidemia (p=0.003), hypertension (p=0.005), neck circumference (p=0.02), and BMI (p<0.001), further underscores its role as a DR and PDR risk amplifier. Our study aligns with the results of Kaswan et al. and Storgaard et al. in this context [16,18]. These factors, prevalent in our high-risk OSA cohort, likely exacerbate retinal hypoxia and vascular damage, particularly in PDR, where 57.14% of high-risk OSA patients were affected. Although using ESS only for scoring the risk of sleep apnea may be considered a limiting factor, it does offer valuable insights in a busy DR clinic. While statistical insignificance tempers causality claims, the clinical signal is clear. Screening for OSA in DR clinics could identify patients at elevated risk for severe retinopathy. Banerjee et al. similarly posited that hypoxemia drives retinal outcomes [13], and our data suggest this effect is magnified in PDR, where neovascularization reflects advanced ischemic stress.

The practical implications of our findings are significant. By using ESS to triage high-risk OSA patients for targeted PSG, we propose a scalable screening approach for DR clinics where routine PSG is impractical. The 57.14% prevalence of PDR in high-risk OSA patients highlights a critical subgroup warranting early intervention, such as CPAP, which Altaf et al. showed reduces PDR severity [10]. While our small PSG sample limits definitive conclusions, it positions this study as a pilot for larger, longitudinal research to confirm the role of OSA in DR and PDR risk. Integrating OSA screening into DR management in India's resource-constrained context could mitigate vision loss, particularly for PDR, where high-risk OSA appears most impactful.

## Conclusions

This study observed a trend toward a higher prevalence of diabetic retinopathy, including proliferative diabetic retinopathy, among individuals at higher risk for obstructive sleep apnea. Although these associations did not reach statistical significance, the findings suggest a possible relationship that may be mediated by intermittent hypoxia and metabolic dysregulation. Screening for obstructive sleep apnea may have potential value in identifying diabetic individuals who could be at increased risk of retinopathy; however, this requires confirmation. The observed trends related to the novel AHI-sleep parameter highlight the need for larger, well-designed studies to further explore the role of obstructive sleep apnea as a potentially modifiable factor in the development and progression of diabetic retinopathy, particularly in the Indian diabetic population.
